# Complete plastid genome of the Chinese medicinal herb *Paeonia obovata* subsp. *Willmottiae* (Paeoniaceae): characterization and phylogeny

**DOI:** 10.1080/23802359.2020.1716641

**Published:** 2020-01-24

**Authors:** Mingying Zhang, Yimin Li, Jing Gao, Ying Chen, Yonggang Yan, Yuqu Zhang, Yang Luo, Gang Zhang

**Affiliations:** a College of Pharmacy, Shaanxi Qinling Application Development and Engineering Center of Chinese Herbal Medicine, Shaanxi University of Chinese Medicine, Xi’an, China;; b Key Laboratory for Plant Diversity and Biogeography of East Asia, Kunming Institute of Botany, CAS, Kunming, China

**Keywords:** Plastid genome, *Paeonia obovata* subsp. *Willmottiae*, phylogeny, medicinal plant

## Abstract

The plastid genome (plastome) of the endemic Chinese medicinal herb *Paeonia obovata* subsp. *Willmottiae* (Paeoniaceae) was sequenced and investigated in this study. The complete plastome is 152,713 bp in length with the typical quadripartite structure, which consists of a large single-copy region (LSC, 84,419 bp), a small single-copy region (SSC, 16,982 bp), and a pair of inverted repeat regions (IRs, 25,656 bp). The overall GC content is 33.2%, and the IR regions are more GC rich (43.2%) than the LSC (36.7%) and SSC (32.8%) regions. A total of 114 unique genes, including 79 protein-coding genes, 31 tRNAs, and four rRNAs were identified. Phylogenetic reconstruction based on complete plastome sequences demonstrated that *P. obovata* subsp. *Willmottiae* is phylogenetically closest to *P. obovata*.

*Paeonia obovata* subsp. *Willmottiae* is a perennial herb belonging to the peony family (Paeoniaceae), which is endemic to China and mainly confined to the mountain area around the Qinling Range (Hong, Pan, Rao, 2001; Hong 2010). Morphologically, it is distinctly different from other *Paeonia* species by leaves densely hirsute or pubescent on the lower surface (Hong, Pan, Turland 2001). The root of *P. obovata* subsp. *Willmottiae* has medicinal value and used as Radix Paeoniae Rubra in some local areas (Editorial Committee of Chinese Materia Medica 1999). To get a better understanding of the characterization of the plastome of *P. obovata* subsp. *Willmottiae* and to uncover its phylogenetic position in *Paeonia*, the complete plastome was sequenced and investigated in this study.

Fresh leaves of *P. obovata* subsp. *Willmottiae* were collected from the Red River Valley Forest Park (Mei County, Shaanxi, China; 34°6′22″N, 107°44′54″E). Voucher specimen (ZMY3) was deposited in the herbarium of traditional Chinese Medicine, Shaanxi University of Chinese Medicine. Total genomic DNA was extracted utilizing the Plant Genomic DNA Kit (TIANGEN) and then used for library preparation. Paired-end sequencing (2 × 150 bp) was performed on the Illumina HiSeq X platform at the Beijing Genomics Institute (BGI) in Shenzhen, China. *De novo* assembly was conducted using GetOrganelle pipeline described by Jin et al. (2018). The assembled circular plastome was then annotated by Plastid Genome Annotator (PGA; Qu et al. 2019) with the published plastome of *P. obovata* (NC_026076) as reference, coupled with manual correction in Geneious 8.02. Transfer RNAs (tRNAs) were confirmed by their specific structure predicted by tRNAscan-SE (Lowe and Chan 2016). Maximum likelihood (ML) phylogenetic analysis among *P. obovata* subsp. *Willmottiae* and other 12 published *Paeonia* species (Figure 1) (Zhang et al. 2016; Bai et al. 2018; Guo et al. 2018; Li et al. 2018; Lee et al. 2019; Zhou et al. 2019; Chen et al. 2019; Zhang et al. in press) was performed based on their complete plastome sequences, under the GTR + G model with 1000 rapid bootstrap replicates, using RAxML Version 8 (Stamatakis 2014). *Bergenia scopulosa* (Saxifragaceae) and *Coptis chinensis* (Ranunculaceae) were selected as outgroup representatives.

**Figure 1.**
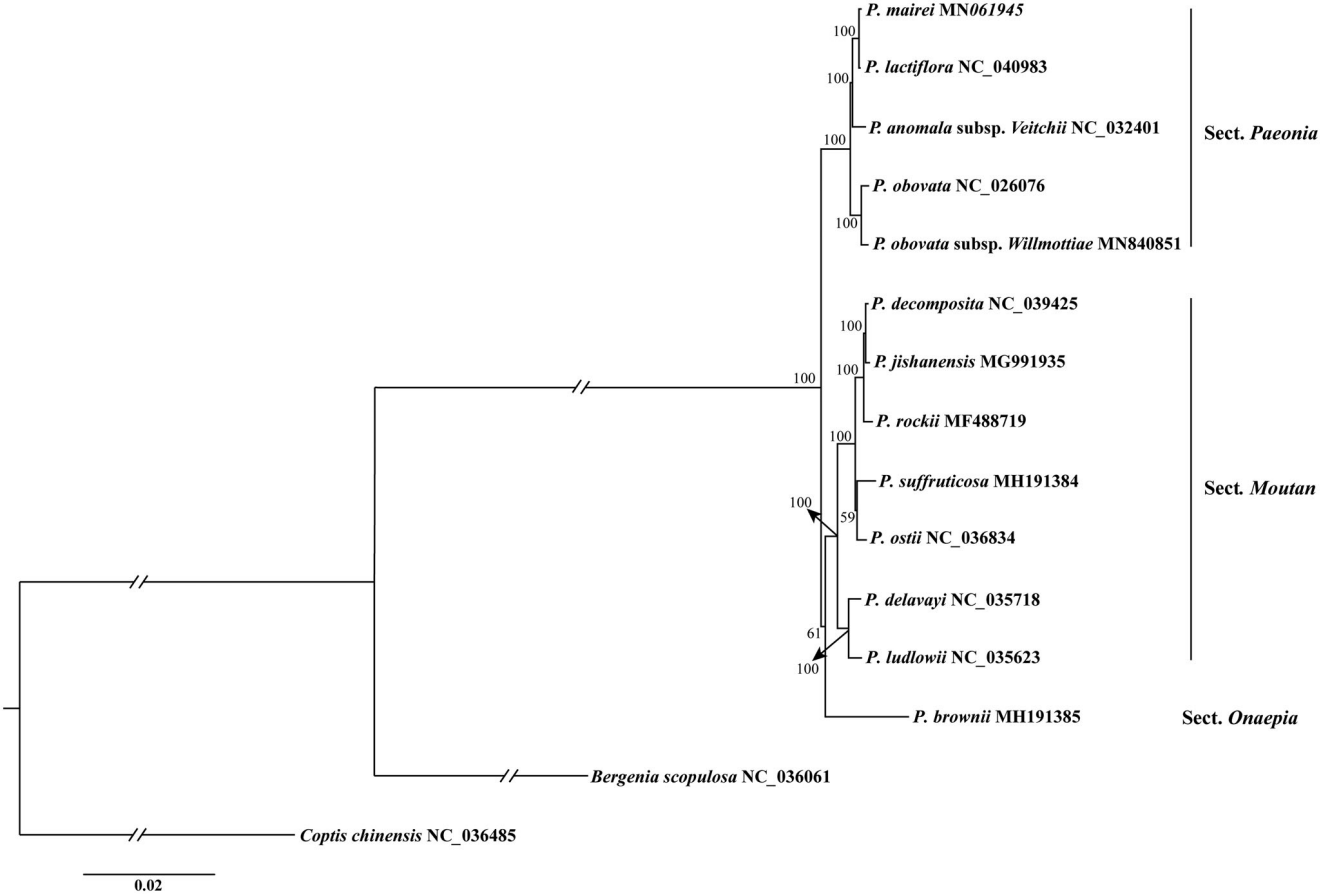
Maximum likelihood phylogenetic tree of 13 *Paeonia* species inferred based on dataset of the complete plastome sequences. Numbers beside each node are bootstrap values.

The complete plastome of *P. obovata* subsp. *Willmottiae* (GenBank accession number: MN840851) displayed the typical quadripartite structure and conserved organization as most angiosperm plastid genomes. It is 152,713 bp in length with an 84,419 bp large single-copy region (LSC), a 16,982 bp small single-copy region (SSC), and a pair of 25,656 bp inverted repeat regions (IRs), and the overall GC content is 33.2%. A total of 114 unique genes, including 79 protein-coding genes, 31 tRNAs, and four rRNAs were identified. The phylogenetic tree showed that all 13 *Paeonia* species forming a monophyletic group supported by a 100% bootstrap value, and which could be divided into three subclades, i.e. Sect. *Paeonia*, sect. *Onaepia* and sect. *Moutan*, corresponding to the classification of *Paeonia* proposed by Stern (1946) and by Hong (2010). *Paeonia obovata* subsp. *Willmottiae* was inferred phylogenetically closest to *P. obovate* and belonging to Sect. *Paeonia* (Figure 1).
